# Predicting Tyrosine Kinase Inhibitor Treatment Response in Stage IV Lung Adenocarcinoma Patients With EGFR Mutation Using Model-Based Deep Transfer Learning

**DOI:** 10.3389/fonc.2021.679764

**Published:** 2021-07-20

**Authors:** Runping Hou, Xiaoyang Li, Junfeng Xiong, Tianle Shen, Wen Yu, Lawrence H. Schwartz, Binsheng Zhao, Jun Zhao, Xiaolong Fu

**Affiliations:** ^1^ School of Biomedical Engineering, Shanghai Jiao Tong University, Shanghai, China; ^2^ Department of Radiation Oncology, Shanghai Chest Hospital, Shanghai Jiao Tong University, Shanghai, China; ^3^ The First Affiliated Hospital of USTC, Division of Life Sciences and Medicine, University of Science and Technology of China, Hefei, China; ^4^ Division of Health Care, Tencent, Shenzhen, China; ^5^ Department of Radiology, Columbia University Irving Medical Center, New York, NY, United States

**Keywords:** deep learning—convolutional neural networks, computed tomography, lung cancer, transfer learning, epidermal growth factor receptor mutation

## Abstract

**Background:**

For stage IV patients harboring EGFR mutations, there is a differential response to the first-line TKI treatment. We constructed three-dimensional convolutional neural networks (CNN) with deep transfer learning to stratify patients into subgroups with different response and progression risks.

**Materials and Methods:**

From 2013 to 2017, 339 patients with EGFR mutation receiving first-line TKI treatment were included. Progression-free survival (PFS) time and progression patterns were confirmed by routine follow-up and restaging examinations. Patients were divided into two subgroups according to the median PFS (<=9 months, > 9 months). We developed a PFS prediction model and a progression pattern classification model using transfer learning from a pre-trained EGFR mutation classification 3D CNN. Clinical features were fused with the 3D CNN to build the final hybrid prediction model. The performance was quantified using area under receiver operating characteristic curve (AUC), and model performance was compared by AUCs with Delong test.

**Results:**

The PFS prediction CNN showed an AUC of 0.744 (95% CI, 0.645–0.843) in the independent validation set and the hybrid model of CNNs and clinical features showed an AUC of 0.771 (95% CI, 0.676–0.866), which are significantly better than clinical features-based model (AUC, 0.624, P<0.01). The progression pattern prediction model showed an AUC of 0.762(95% CI, 0.643–0.882) and the hybrid model with clinical features showed an AUC of 0.794 (95% CI, 0.681–0.908), which can provide compensate information for clinical features-based model (AUC, 0.710; 95% CI, 0.582–0.839).

**Conclusion:**

The CNN exhibits potential ability to stratify progression status in patients with EGFR mutation treated with first-line TKI, which might help make clinical decisions.

## Introduction

Non-small cell lung cancer (NSCLC) has the highest mortality both in United States and China (1, 2), of which lung adenocarcinoma accounts for about 50%. For stage IV lung adenocarcinoma patients harboring EGFR mutations, tyrosine kinase inhibitor (TKI) is recommended to be the first-line treatment modality especially for Asian patients with a relatively higher possibility of EGFR mutations (3). First-line TKI treatment could achieve the median progression-free survival (PFS) of approximately 10 months and a response rate of about 70% (4, 5). However, the disease inevitably progresses owing to acquired resistance to TKI treatment after a period of response. Because of inter-patient and inter-lesion heterogeneity, PFS and progression pattern of first-line TKI treatment are heterogeneous between patients. Different PFS and progression pattern determines different subsequent treatment strategy. For example, it is helpful to increase the PFS and even overall survival (OS) of TKI treatment by the addition of local ablative therapy for patients with favorable PFS and oligoprogression (6) and the enhancement of systematic therapy for patients with poor PFS and systematic progression (7). Therefore, accurate prediction of PFS and progression pattern of first-line TKI treatment is of great significance to the subsequent clinical decision making.

Nowadays, the prediction of PFS and progression pattern of TKI treatment in clinical practice is mainly based on the conventional information such as patient demographics, pathology, and genetics. Nevertheless, these features are low-dimensional with limited representational ability, which may lead to unsatisfactory accuracy. Recently, medical imaging has been widely used to help clinicians for decision making according to some morphological features about the tumor. However, these subjective and qualitative morphological features often result in low inter-observer agreement and limited accuracy. Thus, a more objective and quantitative method to accurately predict PFS and progression patterns of first-line TKI treatment is urgently needed.

Convolutional neural network (CNN) is an artificial intelligence algorithm with the capability to excavate the underlying biological information from medical imaging. Compared with the traditional feature engineering, CNN has great advantages in automatically extracting the latent deep representative features and developing robust end-to-end prediction models. It has been recently utilized in various medical domains with satisfactory results (8). In thoracic oncology, CNN could distinguish malignant pulmonary nodules (9), identify pathological types of lung cancer (10, 11), detect driven oncogene status, and other tasks (12–14) using chest CT images. Therefore, we decided to develop a CNN model to predict PFS and progression patterns of first-line TKI treatment of lung adenocarcinoma patients based on chest CT images.

For the training of CNN, the weights of network are often randomly initialized and then updated under the supervision of image labels, which is called “training from scratch.” This method requires large amounts of data to learn the huge number of CNN parameters. However, in this study, as the number of patients harboring EGFR mutations and treated with first-line TKI is limited, this training strategy may cause overfitting of the CNN model and lead to poor generalization performance. Thus, how to train the CNN network with limited data is the major concern of this study.

Transfer learning is a technique that can help overcome the problem of insufficient training data. Researches have shown that the pre-trained weights and features from one domain are transferable to another domain with similar characteristics (15, 16). In this study, considering the available data are limited, we decided to train the CNN network with deep transfer learning using pretrained CNN models. The basic pretrained CNN model has been developed to distinguish benign and malignant pulmonary nodules in a large data set (with 8472 samples). Then, in light of the large dissimilarity between distinguishing pulmonary nodules (in early-stage patients) and predicting PFS and progression patterns (in stage IV patients), we added the domain of detecting EGFR mutations of stage IV patients for fine-tuning of the basic model. Afterward, this fine-tuned model for EGFR mutation prediction was further utilized to help train the progression prediction models.

Overall, this study aims to develop and validate a CNN model with model-based deep transfer learning to predict the PFS and progression patterns of first-line TKI treatment of lung adenocarcinoma patients based on pretherapy CT images. The pretrained model in source domain based on large data set would help the CNN model in target domain be better trained with limited data.

## Materials and Methods

### Study Design

**Figure 1:**
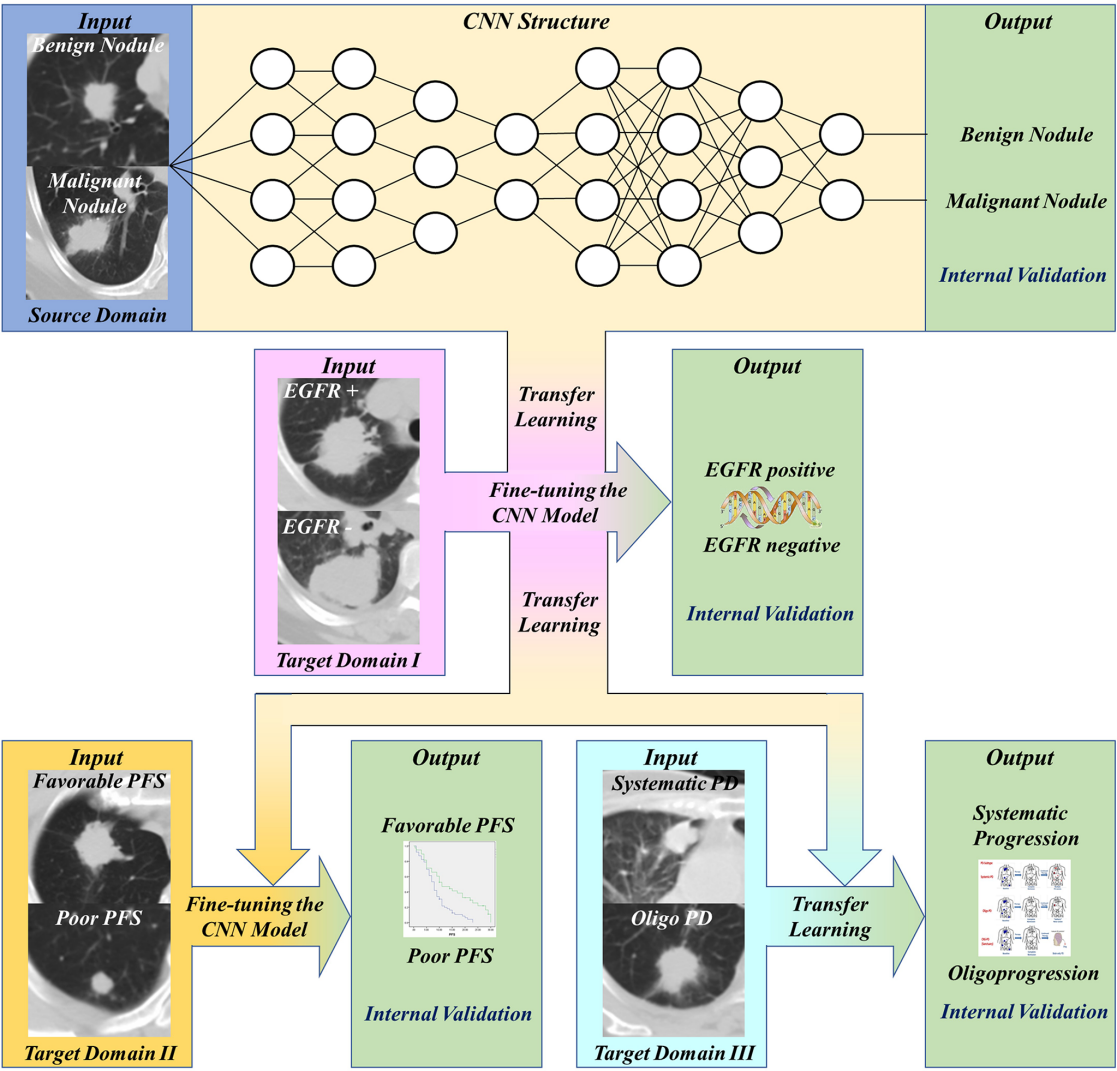
Workflow of our work. First, transfer and finetune the basic pulmonary nodule recognition model to EGFR prediction. Then, transfer the EGFR recognition model to PFS prediction and progression patterns prediction.

This study was approved by Shanghai Chest Hospital, Shanghai Jiaotong University. Ethical approval (ID: KS 1716) was obtained for the use of the CT images and clinical information of patients. Informed consent was waived for the respective nature of the study. The study design was illustrated in **Figure 1**. The basic CNN model was previously constructed by the domain of distinguishing malignant and benign pulmonary nodules. Then, this basic model was fine-tuned by the domain of detecting EGFR mutation of lung adenocarcinoma, and then transferred to predict the PFS and progression patterns of TKI treatment.

### Patients

We retrospectively analyzed patients receiving first-line TKI treatment from 2013 to 2017 in Shanghai Chest Hospital. The inclusion criteria: a. Patients were diagnosed with stage IIIB and IV lung adenocarcinoma harboring EGFR mutations; b. Patients received first-line TKI treatment. c. The smoking history should be clear. d. Patients should undertake completed staging examination to confirm the clinical stage. e. The pulmonary nodules should be solid with the max diameter over 0.8 cm. f. Patients should receive completed follow-up every 3 months to confirm the accurate PFS and restaging examination for judging progression patterns. The exclusion criteria: a. Patients with non-adenocarcinoma; b. Patients without completed staging examinations to confirm the clinical stages of IIIB and IV. c. Patients did not undertake routine follow-up and restaging examinations to confirm the accurate PFS and progression patterns. PFS was defined from the start of TKI treatment to first progression or last follow-up date. For metastatic pattern at initial diagnosis, systemic metastasis was defined as over five metastatic sites or over three organs, and oligometastasis was less than five metastatic sites within three or fewer organs. The progression pattern was classified into oligoprogression and systematic progression. The systematic progression was defined as multi-sites progression, which may include both new metastatic sites, as well as regrowth in previously responsive sites of disease. The oligoprogression was defined to CNS progression without leptomeningeal progression and extra-cerebral progression four or less sites. All the enrolled patients were assigned into training set and validation set randomly.

### CT Image Acquisition and Preprocessing

Chest CT scans were taken with voltage from 120 kV to 140 kV, current 170 mA, scan layer thickness 5 mm, and spatial resolution about 1 mm using Brilliance 64 CT from PHILIPS. Tumors were manually segmented by an experienced radiologist (window level -400 and window width 1600) on the platform Pinnalce2 for Varian^®^. The radiologist was asked to only delineate a rough region of interest covering nodules. Linear interpolation was applied to the original CT images to get isotropic images (1 mm × 1 mm × 1 mm). Image patches were cropped from the interpolated images centered as the tumor.

### Model Development and Evaluation

We established binary classifiers using three dimensional CNN (3D CNN) to distinguish patients with different PFS and progression patterns. Model-based deep transfer learning was utilized to train the CNN more effectively. The 3D CNN was pre-trained on a source task and then the weights of some layers or features were transferred to the target domain task. Moreover, parameter fine-tuning was used to retrain the network on the task of PFS prediction. The workflow of our work was shown in **Figure 1**. Based on the basic model for benign and malignant pulmonary nodules recognition, we use transfer learning and fine-tuning to develop the EGFR classification model, then further transferred this EGFR classification model to PFS and progression patterns prediction.

### Establishment and Fine-Tuning of the Basic Models

The structure of the basic model for nodule recognition (CNN_BM_) was based on a 3D residual network with prior attention. The residual network can effectively tackle the vanishing gradient problem in deep neural networks. The inputs of the network include both the CT image patch and corresponding mask, which cover the region of interest (ROI), to make the network focusing on image pixels within the ROI. As the sample size was large for training (8,472 samples), the basic model was trained from scratch. The details of the basic CNN architecture can be found in **Supplementary Table S1**.

An EGFR classification model (CNN_EGFR_) was constructed through fine-tuning of the pre-trained basic model. The fine-tuning process makes the network more applicable for the IV stage patients, and some latent EGFR mutation-related features can be learned, which may be helpful for the PFS prediction after TKI treatment.

### Establishment of PFS Prediction by Transfer Learning

For PFS prediction, patients with favorable PFS (>9 months) were regarded as positive samples with label 1, and those with poorer PFS (<=9 months) were negative samples with label 0. To develop the PFS prediction model, we transfer and fine-tune the pre-trained 3D CNN model in two steps. First, we freeze the top- layers’ parameters and only train the fully connected layers with a larger initial learning rate of 1e-2. After 10 epochs training, we unfreeze the frozen layers and fine-tune the whole network with a smaller initial learning rate of 1e-4. The CNN_EGFR_ was also trained in this way based on the CNN_BM_. To evaluate the effect of transfer learning and the influence of domain difference, we compare different 3D CNN PFS prediction models respectively fine-tuned from the CNN_EGFR_, CNN_BM_, and trained from scratch. Furthermore, clinical features, such as age, sex, smoking, clinical stages, and molecular pathology status, were fused with 3D CNN model by logistic regression for better prediction. Tensorflow (tensorflow.org) was used for network training.

### Establishment of Progression Pattern Prediction by Transfer Learning

For progression patterns prediction, patients with systematic progression were regarded as positive samples, and patients with oligoprogression were negative samples. To develop the progression patterns prediction model, we use the pre-trained CNN_EGFR_ as feature extractor and then construct classifiers. The deep features (dimensional feature vectors, 128) were extracted from the last layer before the outputs. After feature extraction, univariate feature selection and recursive feature elimination were used to select features, then decision tree, random forest, and K-Nearest Neighbor classifiers were constructed to realize the final prediction. Furthermore, because T stage and metastasis status are significant factors related to patients’ progression patterns, we used the two factors to build a logistic regression model as the baseline. Finally, this basic model and the image-based model were fused to develop the hybrid prediction model. The algorithms were implemented with scikit-learn (scikit-learn.org) in python.

### Statistical Analysis

Statistical analysis was conducted in R software (Rproject.org). Fisher’s exact test, Wilcoxon test, and chi-square test were used to compare the differences of clinical features between training and validation groups. For model evaluation, the receiver operating characteristic (ROC) curve and AUC were used to describe model performance, and DeLong (17) test was used to pairwise compare the difference of two ROCs. Kaplan-Meier survival curves of the subgroups stratified by our model (favorable/poor PFS) were plotted, and log-rank test was used to compare difference of two KM curves. P value less than 0.05 was considered as significant.

## Results

### Patient Characteristics

We retrospectively analyzed 339 patients for the creation of PFS prediction model. Patients were randomly divided into training group (70.5%) and validation group (29.5%). No significant difference was found between the two groups in terms of all clinical characteristic (**Table 1**). The median PFS of total patients was 9 months. There were 169, 160, and 10 patients harboring EGFR exon 19, exon 21, and double site mutation, respectively. At Cox proportional hazard regression, all the clinical characteristics, including age, gender, smoking status, clinical stage, and EGFR mutation site, were not prognostic for PFS. After excluding patients without confirmed progression pattern, totally 255 patients were enrolled for the establishment of progression pattern prediction model. The detailed characteristics of patients were shown in **Table 2**. For the metastatic pattern in the initial diagnosis, there were 186 (72.9%) and 55 (21.6%) patients demonstrating systematic metastasis and oligometastasis, respectively. While at acquired resistance to TKI, 153 (60%) and 102 (40%) displayed systematic progression and oligoprogression, respectively. At multivariate logistic regression, T stage (OR=1.70, p<0.001) and metastatic pattern (OR=3.29, p=0.006) were recognized to be related with progression pattern.

**Table 1:** Comparison of clinical features in patients with PFS information.

Clinical Features	Training group (n = 239)	Validation group (n = 100)	p value
**Age**			
Median (Range)	61 (33-84)	61 (26-82)	t-test p=0.217
**Gender (n%)**			
Male	97 (40.6)	32 (32.0)	Pearson χ^2^ Test p=0.143
Female	142 (59.4)	68 (68.0)	
**Smoking History**			
Yes	55 (23.0)	17 (17.0)	Pearson χ^2^ Test p=0.265
No	184 (77.0)	83 (83.0)	
**PFS (months)**			
Median	9	9	Log-rank Test p=0.265
≤9 months	138 (57.7)	56 (56.0)	Pearson χ^2^ Test p=0.810
>9 months	101 (42.3)	44 (44.0)	
**Clinical Staging**			
IIIA	8 (3.3)	3 (3.0)	Mann-Whitney Test p=0.989
IIIB	22 (9.2)	7 (7.0)	
IV	209 (87.5)	90 (90.0)	
**EGFR mutation site**			
19del	121 (50.6%)	48 (48%)	Pearson χ^2^ Test p=0.346
21L858R	113 (47.3%)	47 (47%)	
Double Site	5 (2.1%)	5 (5%)	

**Table 2:** Comparison of clinical features in patients with progression patterns information.

Clinical Features	Training group (n = 195)	Validation group (n = 60)	p value
**Age**			
Median (range)	61 (26–81)	59 (35–84)	t-test p=0.777
**Gender (n%)**			
Male	77 (39.5)	20 (33.3)	Pearson χ^2^ test p=0.737
Female	118 (60.5)	40 (66.7)	
**Smoking history**			
Yes	156 (80.0)	49 (81.7)	Pearson χ^2^ test p=0.776
No	39 (20.0)	11 (18.3)	
**PFS (months)**			
Median	8	11	Log-rank test p=0.131
**T Stage**			
T1	38 (19.5%)	11 (18.3%)	Mann-Whitney test p=0.865
T2	41 (21.0%)	12 (20%)	
T3	16 (8.2%)	6 (10.0%)	
T4	100 (51.3%)	31 (51.7%)	
**Metastasis pattern at initial diagnosis**			
Oligometastasis	26 (13.3%)	13 (21.7%)	Pearson χ^2^ test p=0.117
Systematic metastasis	169 (86.7)	47 (78.3)	
**Progression pattern**			
Oligoprogression	77 (39.5)	25 (41.7)	Pearson χ^2^ test p=0.763
Systematic progression	118 (60.5)	35 (58.3)	
**EGFR mutation site**			
19del	105 (53.8%)	33 (55%)	Pearson χ^2^ test p=0.756
21L858R	83 (42.6%)	26 (43.3%)	
Double site	7 (3.6%)	1 (1.7%)	

### Structure and Performance of Basic Models

The basic model we developed to distinguish malignant pulmonary nodules achieved good performance with a high AUC value of 0.932 (95% CI, 0.924–0.947). When utilizing the domain of detecting EGFR mutation for fine-tuning, the AUC value of the model was 0.863 (95% CI, 0.763–0.897) (**Supplementary Figures S2, S3**).

### PFS Prediction

**Figure 2:**
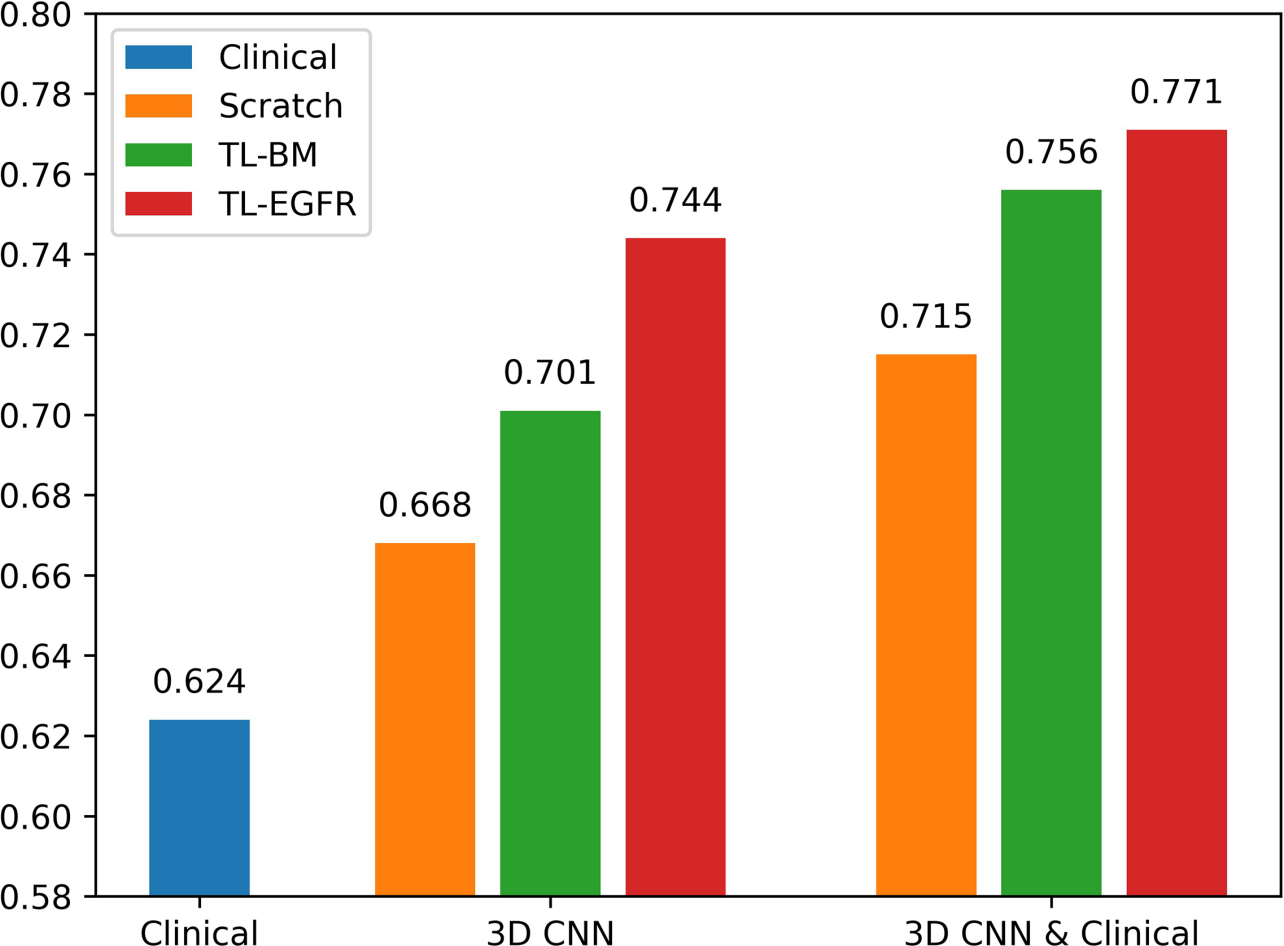
AUCs of each PFS prediction models in the validation group. The blue ones correspond to the clinical alone model. The orange, green, and red ones correspond to CNN model trained from scratch, transferred from nodule, and transferred from EGFR classification models, receptively.

**Figure 3:**
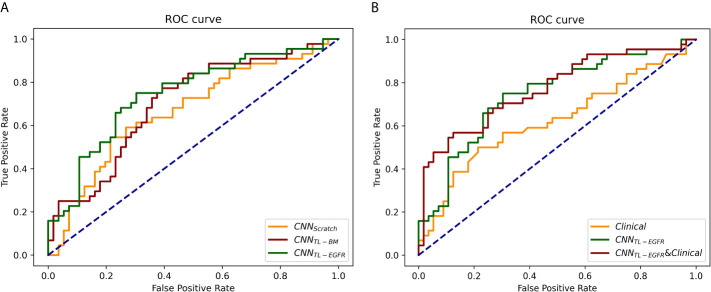
ROCs of 3D CNN models for the prediction of PFS in the validation group. **(A)** The ROCs of 3D CNN model trained from scratch, using transfer learning based on nodule or EGFR classification models. The corresponding AUCs were 0.668, 0.701, and 0.744, receptively. **(B)** The ROCs of only using clinical features, 3D CNN (using transfer learning based on EGFR classification model), and the combination of 3D CNN and clinical features, and the corresponding AUCs were 0.624, 0.744, and 0.771.

The 3D-CNN model trained from scratch (CNN_Scratch_), transferred directly from the basic model (CNN_TL-BM_) and from the EGFR mutation fine-tuned model (CNN_TL-EGFR_) showed AUCs of 0.668 (95% CI, 0.559–0.776), 0.701 (95% CI, 0.598–0.805), and 0.744 (95% CI, 0.645–0.843) in the validation group, respectively (**Figures 2**, **3A**). The 3D CNN model with EGFR classification fine-tuning achieved better performance than the model directly transferred from the basic model, mainly because the domain difference between EGFR classification and PFS stratification is smaller than the difference between nodule classification and PFS stratification. Moreover, after adding the clinical features, the corresponding fusion model’s performance improved to 0.715 (95% CI, 0.614–0.816), 0.756 (95% CI, 0.659–0.854), and 0.771 (95% CI, 0.676–0.866), respectively (**Figure 2** and **Table 3**). As shown in **Figure 3B**, the best 3D CNN model (CNN_TL-EGFR_) performed better than clinical features-based model (AUC, 0.744 vs 0.624). Furthermore, the fusion model (CNN_TL-EGFR_ and Clinical) achieved significantly better performance than the clinical model alone (P=0.008).

**Table 3:** Performance of different PFS prediction models in the validation group.

Models	CNN_TL-EGFR_	CNN_TL-BM_	CNN_Scratch_
AUC	0.744	0.701	0.668
95% CI	0.645 to 0.843	0.598 to 0.805	0.559 to 0.776
Threshold	0.449	0.379	0.490
Accuracy	72.0%	68.0%	68.0%
Sensitivity	75.0%	77.3%	54.5%
Specificity	69.6%	60.7%	78.6%
**Models**	**CNN_TL-EGFR_ and Clinical**	**CNN_TL-BM_ and Clinical**	**CNN_Scratch_ and Clinical**
AUC	0.771	0.756	0.715
95% CI	0.676 to 0.866	0.659 to 0.854	0.614 to 0.816
Threshold	0.575	0.615	0.496
Accuracy	74.0%	75.0%	70.0%
Sensitivity	56.8%	52.3%	56.8%
Specificity	87.5%	92.9%	80.4%

CNN, convolutional neural network; AUC, area under receiver operating characteristic curve; threshold, threshold at the optimal decision point; CI, confidence interval.

**Figure 4:**
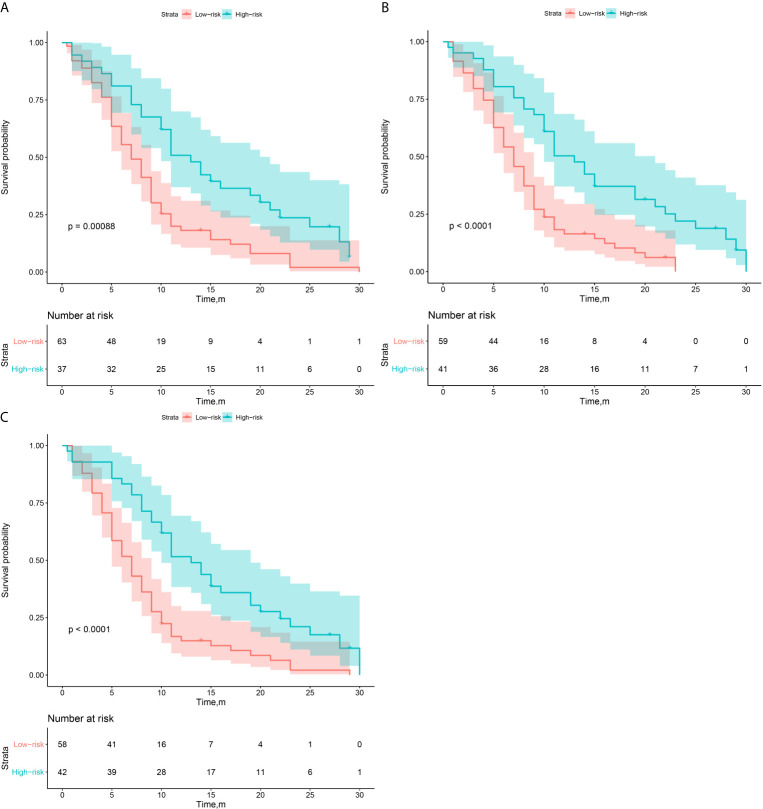
Survival analysis of PFS in low and high risk patients in the validation group. **(A–C)** The CNN_Scratch_ and clinical, CNN_TL-BM_ and clinical, and CNN_TL-EGFR_ and clinical model’s KM curves, respectively.

Then, according to the prediction results of different 3D CNN models, we divided the validation group into high-risk and low-risk subgroups. The optimal cutoff threshold was confirmed by X-tile (18). Based on this, Kaplan-Meier survival curves were plotted respectively in the two subgroups. As shown in **Figure 4**, the CNN_TL-EGFR_ and clinical fusion model achieved the best performance and can significantly distinguish the difference in PFS between the stratified progression subgroups (log-rank test, P<0.001).

### Progression Pattern Prediction

As above-mentioned, the addition of EGFR recognition fine-tuning achieved the highest prediction efficacy among all the 3D-CNN models. Therefore, in the prediction of progression pattern, we utilized transfer learning from the EGFR classification to develop the progression pattern model.

**Figure 5:**
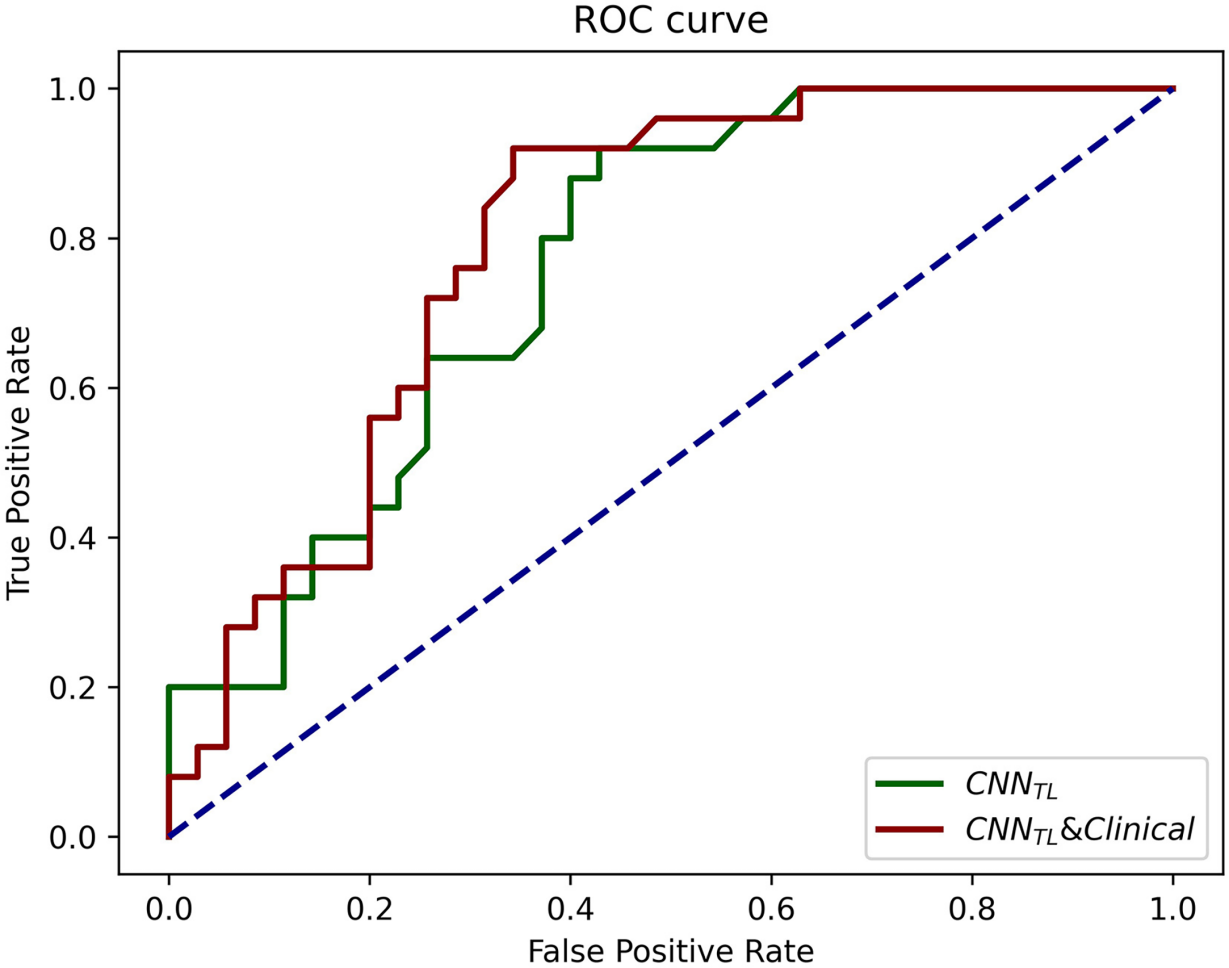
The ROCs of CNN_TL_ (transferred from the EGFR classification) and the CNN_TL_ and clinical models for progression patterns prediction in the validation group. The corresponding AUCs were 0.76 and 0.79.

The progression patterns prediction model transferred from EGFR classification achieved an AUC of 0.762 (95% CI, 0.643–0.882; sensi, 0.92; speci, 0.571). Clinical features-based model achieved an AUC of 0.710 (95% CI, 0.582–0.839; sensi, 0.686; speci, 0.760), and the hybrid model achieved an AUC of 0.794 (95% CI, 0.681–0.908; sensi, 0.92; speci, 0.66). The ROCs of the models in the validation group were shown in **Figure 5**.

## Discussion

In this study, we developed a PFS prediction and a progression pattern prediction model using model-based deep transfer learning based on a pre-trained EGFR classification CNN model. Results show that the hybrid model combining transfer learning-based and clinical features-based model finally achieved satisfactory performance for PFS prediction (AUC = 0.771) and progression pattern prediction (AUC = 0.794). Also, the PFS prediction model can significantly stratify patients with different progression risk after first-line TKI treatment (P < 0.001). Utilization of the established CNN model could instruct clinical practice to individually modify TKI treatment for a better prognosis.

The above results indicate that image-based deep learning can mine more informative features for the prediction of tumor’s biological behavior. Also, the results indicate that 3D CNN trained with model-based deep transfer learning performs better than model training from scratch (AUC, 0.668–0.744), and the smaller the difference between source domain and target domain, the better performance can transfer learning achieve (AUC, 0.701–0.744). Compared with the most widely used transfer learning pretrained on 2D natural image data set (ImageNet) (14, 19), our 3D transfer learning is based on 3D medical image data set, which can not only mine more spatial information but also effectively reduce the domain difference.

A recent study about predicting EGFR-TKI treatment response using CT images (20) used a self-supervised learning-based model called BigBiGAN as a feature extractor, then utilize the extracted features to construct a Cox regression model for distinguishing patients with different progression risk. In comparison, we used a supervised learning based pre-trained model for transfer learning, then utilize the progression label to finetune the model and update the extracted deep features. Compared with the BigBiGAN model trained in self-supervised ways, our pre-trained model trained with EGFR status can learn not only the inherent grayscale-based features but also some implicit biologically related image features. Moreover, because of the small difference between the source domain (classification of EGFR mutation) and target domain (prediction of EGFR-TKI therapy response) of our proposed method, the network can make fully use of the pre-learned features effectively, which can better help the prediction of disease progression.

This research also has several limitations. First, because of the limitation of sample size, this study only realized the simple binary classification of patients’ PFS with the median survival as the cutoff threshold. In the future, we will collect more samples, and further attempt deep Cox regression to realize the end-to-end survival prediction. Second, our hypothesis of the relationship between EGFR mutation and patient’s PFS is that the mutation abundance is thought to be related with the patient’s survival (21). Therefore, we thought the CNN classifying EGFR mutation status will also be able to learn information about the mutation abundance, which may be useful for PFS prediction. However, this mutation abundance information learning was clearly insufficient. In the future, if the mutation abundance information of the EGFR mutation patients can be collected, a more efficient network can be built and further correlate with the patient’s PFS. Finally, the model should be validated in a prospective cohort to confirm its efficacy.

## Conclusion

We developed a deep transfer learning-based PFS prediction and progression pattern prediction model in EGFR mutation patients treated with TKIs. The results showed that the prediction model transferred from EGFR classification can significantly stratify patients with different progression risk after TKI treatment, which may be able to further help the clinical decision making.

## Data Availability Statement

The data sets presented in this article are not readily available because the data sets are privately owned by Shanghai Chest Hospital and are not made public. Requests to access the data sets should be directed to XF, xlfu1964@hotmail.com.

## Ethics Statement

The studies involving human participants were reviewed and approved by Shanghai Chest Hospital. The ethics committee waived the requirement of written informed consent for participation.

## Author Contributions

XF, JZ, RH, and XL contributed to the study concept and design. XL, RH, and TS contributed to acquisition of data. RH, XL, JX, WY, LS, and BZ contributed to analysis and interpretation of data. RH and XL contributed to drafting of the manuscript. The corresponding author had full access to all of the data and took full responsibility for the veracity of the data and the statistical analyses. All authors contributed to the article and approved the submitted version.

## Funding

This work was supported in part by the Major Research Plan of the National Natural Science Foundation of China (Grant No. 92059206), Shanghai Jiao Tong University Medical Engineering Cross Research Funds (Nos. YG2017ZD10 and YG2014ZD05), National Key Research and Development Program (Nos. 2016YFC0905502 and 2016YFC0104608), National Natural Science Foundation of China (No. 81371634), and the Fundamental Research Funds for the Central Universities (WK9110000177).

## Conflict of Interest

Author JX was employed by Tencent.

The remaining authors declare that the research was conducted in the absence of any commercial or financial relationships that could be construed as a potential conflict of interest.

## Abbreviations

3D CNN, three-dimensional convolutional neural network; CI, confidence interval; PFS, progression free survival; TKI, tyrosine kinase inhibitor; ROI, region of interest.
