# Supplementary material for: Predicting Tyrosine Kinase Inhibitor Treatment Response in Stage IV Lung Adenocarcinoma Patients With EGFR Mutation Using Model-Based Deep Transfer Learning
Source: Front Oncol. 2021 Jul 20;11:679764. doi: 10.3389/fonc.2021.679764 (PMC8329710; doi:10.3389/fonc.2021.679764)
Supplement: Supplementary file 1 [file DataSheet_1.docx]

**Supplementary Materials**

**The structure of our 3D CNN**

The structure of our 3D CNN model is shown in Table S1, which consisted of two parts, data fusion and main structure. For the data fusion part, the CT image patch and the corresponding segmentation mask were separately convolved by a kernel of $333$ to obtain channel 1 and 2, respectively. Then the two channels were concatenated together and convolved by a $333$ kernel with stride=2 as the input of the main structure. This operation reduces the original feature map of 71×71×71 to the size of 35×35×35.

For the main structure part, there are four residual blocks, which contains 3, 4, 23 and 3 bottlenecks, respectively. Each of the bottleneck contains the residual and shortcut branches. The residual branch consists of three BN-Relu-Conv modules and the shortcut branch is the addition of the input and residual branch’s output.

**Data augmentation**

As the sample size was limited, we use data augmentation to avoid overfitting. We do online augmentation including rotations and translations. For a given tumor patch and the corresponding mask, they were first translated by one to three voxels in three directions. Then the translated images were randomly rotated by a random angle ranging 0° to 360° around x-, y-, and z-axis.

**Training** **of the nodule classification 3D CNN model**

We used cross-entropy function as loss function and stochastic gradient descent (SGD) to train the model. For the nodule classification network training, the training weights of kernels were initialized by Xavier function. The initial learning rate was 0.1, and it was reduced by 0.96 for each 10 epochs. The maximum iterative epoch was set to 1000. The batch size for each iteration was set to 32.

*Tensorflow* was used for the training of 3D CNN and data augmentation. The hardware was NVIDIA TITAN XP.

**Training of the EGFR classification 3D CNN model**

For the EGFR recognition network training, we transfer and fine-tune the pre-trained nodule classification model in two steps. First, freeze the top-layers’ parameters and only train the fully connected layers with a larger initial learning rate of 1e-2. After ten epochs’ training, unfreeze the frozen layers and fine-tuning the whole network with a smaller initial learning rate of 1e-4. Cross-entropy was used as loss function and SGD was utilized to train the network.

**Training of the 3D CNN model for PFS prediction**

For the PFS prediction network training, we use training from scratch and transfer learning strategy, respectively. Cross-entropy function was utilized as loss function and SGD was used to optimize the loss function. The 3D CNN training from scratch randomly initialized the kernel weights by Xavier function. Learning rate was set to 0.01 and the batch size was 32. While, the 3D CNN fine-tuning from the pre-trained classification model was trained in two steps. First, freeze the top-layers’ parameters and only train the fully connected layers with a larger initial learning rate of 1e-2. After ten epochs’ training, unfreeze the frozen layers and fine-tuning the whole network with a smaller initial learning rate of 1e-4. The batch size was also set to 32.

Table S1. The structure of our 3D CNN model.

| Layer_name | 101-layer 3D Resnet | |
| --- | --- | --- |
| Input | CT_patch | Mask_patch |
| Data fusion | 3*3*3, 1, s=1 | 3*3*3, 1, s=1 |
|  | 3*3*3, 16, s = 2 | |
| Conv1_x | 5*5*5, 64, s = 1 | |
|  | 1*1*1, 32  3*3*3, 32  1*1*1, 128  ×3 | |
| Conv2_x | 1*1*1, 32  3*3*3, 32  1*1*1, 128  ×4 | |
| Conv3_x | 1*1*1, 64  3*3*3, 64  1*1*1, 256  ×23 | |
| Conv4_x | 1*1*1, 128  3*3*3, 128  1*1*1, 512  ×3 | |
|  | Average Pool, fc, softmax | |

**Results of the pretrained nodule classification model**

The nodule classification model (Benign VS Malignant) trained with 8472 patient’s CT images finally achieved a satisfactory AUC of 0.932. (21% for testing). The ROCs of the models were shown in Figure s2.


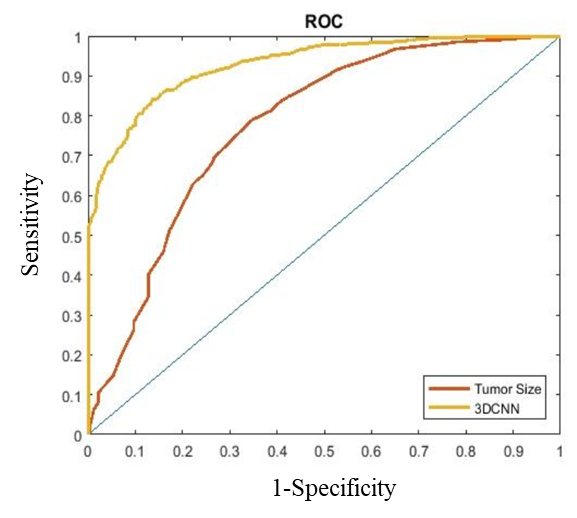


Figure S2. The ROC curves of tumor size baseline and 3D CNN (with priori attention map, PAM) for nodule classification in the testing cohort and the corresponding AUCs were 0.771 and 0.932 , respectively.

**Results of the fine-tuned EGFR mutation classification model**

The EGFR mutation recognition model fine-tuning from the pretrained nodule classification model finally achieved an AUC of 0.863 with 1010 stage IV patient’s CT images (19.8% for testing). The ROCs of the models were shown in Figure S3.


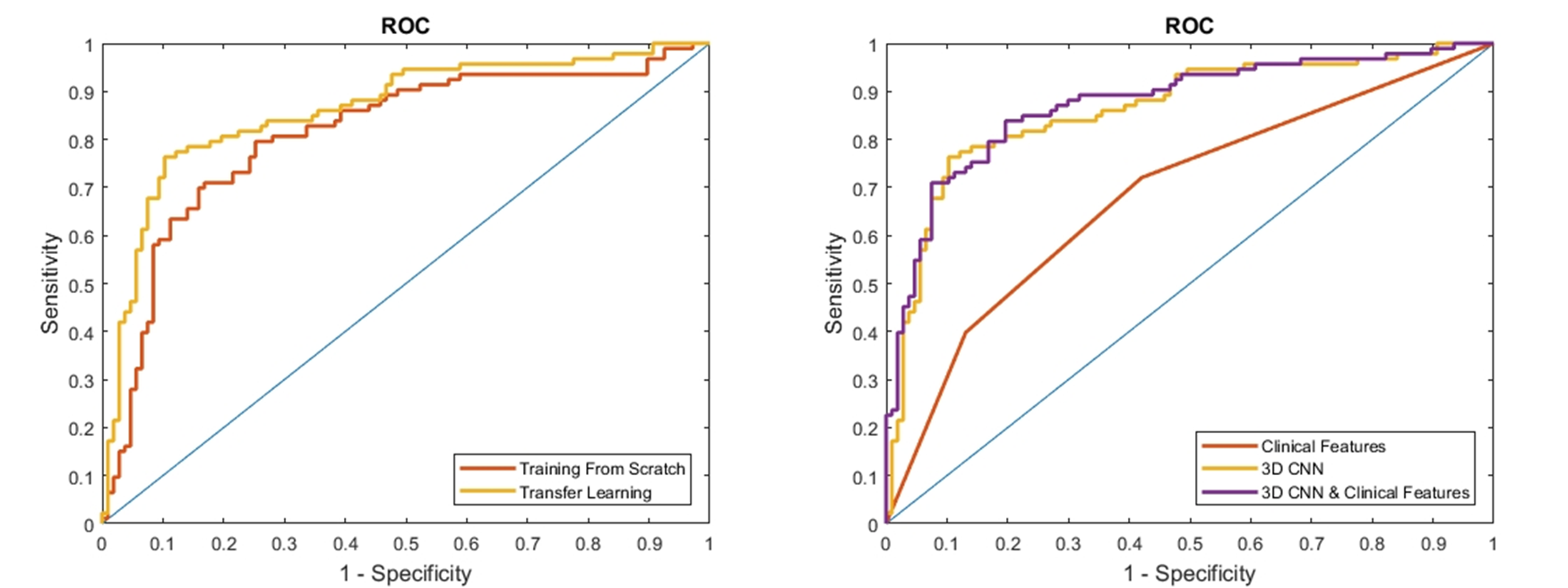


Figure S3. ROCs of 3D CNN models for the detection of EGFR. The left one represents the ROCs of 3D CNN model trained from scratch or using transfer learning based on nodule classification model. The corresponding AUCs were 0.811 and 0.863 (p=0.013). The right one represents the ROCs of only using clinical features, 3D CNN, and the combination of 3D CNN & clinical features, and the corresponding AUCs are 0.686, 0.863, and 0.875, receptively.
